# An EMT-Related Gene Signature for Predicting Response to Adjuvant Chemotherapy in Pancreatic Ductal Adenocarcinoma

**DOI:** 10.3389/fcell.2021.665161

**Published:** 2021-04-30

**Authors:** Zengyu Feng, Kexian Li, Jianyao Lou, Yulian Wu, Chenghong Peng

**Affiliations:** ^1^Department of General Surgery, Pancreatic Disease Center, Ruijin Hospital, Shanghai Jiao Tong University School of Medicine, Shanghai, China; ^2^Research Institute of Pancreatic Diseases, Shanghai Jiao Tong University School of Medicine, Shanghai, China; ^3^Department of General Surgery, The Second Affiliated Hospital, School of Medicine, Zhejiang University, Hangzhou, China

**Keywords:** PDAC, EMT, adjuvant chemotherapy, sensitivity, disease-free survival, risk score, prognostic signature

## Abstract

**Background:**

For pancreatic ductal adenocarcinoma (PDAC) patients, chemotherapy failure is the major reason for postoperative recurrence and poor outcomes. Establishment of novel biomarkers and models for predicting chemotherapeutic efficacy may provide survival benefits by tailoring treatments.

**Methods:**

Univariate cox regression analysis was employed to identify EMT-related genes with prognostic potential for DFS. These genes were subsequently submitted to LASSO regression analysis and multivariate cox regression analysis to identify an optimal gene signature in TCGA training cohort. The predictive accuracy was assessed by Kaplan–Meier (K-M), receiver operating characteristic (ROC) and calibration curves and was validated in PACA-CA cohort and our local cohort. Pathway enrichment and function annotation analyses were conducted to illuminate the biological implication of this risk signature.

**Results:**

LASSO and multivariate Cox regression analyses selected an 8-gene signature comprised DLX2, FGF9, IL6R, ITGB6, MYC, LGR5, S100A2, and TNFSF12. The signature had the capability to classify PDAC patients with different DFS, both in the training and validation cohorts. It provided improved DFS prediction compared with clinical indicators. This signature was associated with several cancer-related pathways. In addition, the signature could also predict the response to immune-checkpoint inhibitors (ICIs)-based immunotherapy.

**Conclusion:**

We established a novel EMT-related gene signature that was capable of predicting therapeutic response to adjuvant chemotherapy and immunotherapy. This signature might facilitate individualized treatment and appropriate management of PDAC patients.

## Introduction

Pancreatic ductal adenocarcinoma (PDAC) is a highly malignant and devastating disease with a 5 years survival rate not exceeding 10% and its incidence increases about 1% per year in the United States (Siegel et al., 2021). The dismal outcome of this malignancy is primarily due to a frequently late diagnosis, mostly at the metastatic and unresectable stage, and the notorious chemoresistance (Kamisawa et al., 2016). Surgery combined with adjuvant chemotherapy is the established therapy option for resectable PDAC patients (Mizrahi et al., 2020). Unfortunately, early postoperative recurrence in most patients caused by the inherent resistance to adjuvant chemotherapy limits the dramatic improvement of patient survival (Kleeff et al., 2016). Currently, adjuvant chemotherapy is administrated empirically, and individual survival benefit of this approach is still questionable. In PDAC patients, the clinical benefit response rates to regimens of chemotherapy are extremely low (Han et al., 2021). Non-responding patients are likely to suffer a variety of adverse events including asthenia and nausea (Phua et al., 2018). These intractable issues have motivated a number of groups to identify robust biomarkers that can predict therapeutic response to chemotherapy in PDAC patients (Kyrochristos et al., 2018).

As precision medicine has shown promising signs, *a priori* prediction of treatment response may facilitate individual management and maximize survival benefit of PDAC patients (Tu et al., 2016). Multiple studies have reported that a treatment-related decrease in serum CA19-9 can predict response to treatment (Xu et al., 2018; Aoki et al., 2019; Perri et al., 2020, 2021). Pre-clinical and clinical evidence demonstrates that patients with specific PDAC subtypes response differently to available treatments (Collisson et al., 2011; Aung et al., 2018). Several genes participating in drug uptake and metabolism have emerged as powerful predictors of drug sensitivity (Bird et al., 2017; Raffenne et al., 2019; Okamura et al., 2020). Recently, with the adventure of high throughput sequencing and bioinformatic technology, more and more gene expression signatures have been identified to evaluate drug sensitivity in PDAC (Kaissis et al., 2019; Clayton et al., 2020; Piquemal et al., 2020; Nicolle et al., 2021; Nishiwada et al., 2021).

Epithelial to mesenchymal transition (EMT) program is related to phenotypic conversion of epithelial cells into more aggressive mesenchymal-like cells and suppression of EMT results in enhanced gemcitabine sensitivity in PDAC mice (Zheng et al., 2015). Compelling evidence has proved a strong association between EMT-related gene expression and therapeutic resistance (Shibue and Weinberg, 2017). For instance, Byres et al. constructed a 76 gene signature based on EMT-related genes with satisfactory accuracy in predicting clinical response to EGFR and PI3K inhibitors for patients with non-small-cell lung carcinoma (Byers et al., 2013). These studies suggest EMT represents an under-explored source of credible biomarkers that could be used to predict drug response.

We purposed to establish a model for predicting response to adjuvant chemotherapy based on EMT-related genes in PDAC. We measured the association between EMT-related genes and disease-free survival (DFS), and established an 8-gene signature with excellent predictive performance in both training and validation datasets. Functionally, this signature is closely related to several pathways involved in drug response. Interestingly, we found that this signature also had potential to predict response to immune-checkpoint inhibitors (ICIs). These findings may facilitate personalized treatment and may potentially exempt patients from heavy finical burden and unnecessary adverse effects of overtreatment.

## Materials and Methods

### PDAC Cohorts

Two public PDAC cohorts were included in this study. Among them, TCGA cohort was used as the training set, while PACA-CA cohort was used for external validation. Processed RNA-sequencing data and corresponding clinical data of TCGA cohort were downloaded from TCGA hub at UCSC Xena^1^. In the cases of PACA-CA cohort, normalized RNA-sequencing data and clinical information were retrieved and downloaded from the International Cancer Genome Consortium (ICGC)^2^ database. In each cohort, the following criteria were used to exclude unqualified samples: (a) follow-up time < 1 month; (b) lack of survival and therapeutic data; (c) histopathological type is not PDAC. After a careful review, 99 samples in TCGA cohort and 105 samples in PACA-CA cohort were included in this study. All patients received adjuvant chemotherapy in both cohorts, and detail of chemotherapeutic drugs was only available in TCGA cohort. Patients whose response to chemotherapy is “clinical progressive disease” or “stable disease” were defined as chemotherapy-resistant, while patients whose response to chemotherapy is “complete response” or “partial response” were defined as chemotherapy-sensitive. Given the medium size of the cohorts we used, we additionally verified the EMT signature in our own cohort (Ruijin cohort). 48 PDAC frozen samples were collected as previously reported (Feng et al., 2020).

### Construction of the EMT-Related Gene Signature for DFS Prediction

A total of 1,184 EMT-related genes were obtained from a previous article (Cai et al., 2020). In the TCGA training cohort, EMT-related genes that were significantly associated with DFS were screened using univariate cox regression analysis (*P* < 0.01). Subsequently, LASSO regression combined with multivariate cox regression analyses were used to determine the optimal risk model. The risk score was calculated as follows: Risk score = (coefficient 1 ^∗^ expression value of gene 1) + (coefficient 2 ^∗^ expression value of gene 2) + . + (coefficient X ^∗^ expression value of gene X).

### Predictive Performance of the EMT-Related Gene Signature

Patients in each cohort were classified into low- and high-risk groups based on the medium value of risk scores. Kaplan–Meier (K-M) survival curves were employed to evaluate the DFS differences between low- and high-risk groups. Calibration plots comparing the predicted and observed survival probabilities were performed to assess the predictive accuracy. Receiver operating characteristic (ROC) curves were utilized to compare the efficiency of the signature with that of clinical predictors for DFS prediction. In addition, univariate and multivariate cox regression analyses were utilized to verify the independent prognostic role of the signature.

### Functional Annotation and Pathway Enrichment

Aiming to clarify the biological function of the EMT signature, we conducted Pearson correlation analysis to identify genes whose expression levels were significantly (*P* < 0.05) correlated with risk scores in TCGA training cohort. Top 1,000 positively and negatively correlated genes were, respectively, submitted to Gene Ontology (GO) analysis and The Kyoto Encyclopedia of Genes and Genomes (KEGG) pathway enrichment analysis on DAVID online website (Huang et al., 2007).

### Quantitative Real Time Polymerase Chain Reaction (qRT-PCR)

Reverse transcription and qRT-PCR were performed as previously reported (Feng et al., 2020). The mRNA primer sequences are displayed in Supplementary Table 1.

### Statistical Analysis

The statistical analysis and graphical work were done in the R environment (version 3.5.2). cox regression analyses were conducted by the “survival” package. K-M survival curves with log-rank tests were produced by the “survminer” package. LASSO regression analysis was done by the “glmnet” package. The ROC curves were plotted by the “survivalROC” package. Boxplots were depicted by the “ggpubr” package. Forest plot was derived from the “forestplot” package. Calibration curves were generated from the “rms” package. A two-sided log-rank *P* < 0.05 was considered significant.

## Results

### Construction of the EMT-Related Gene Signature

**FIGURE 1:**
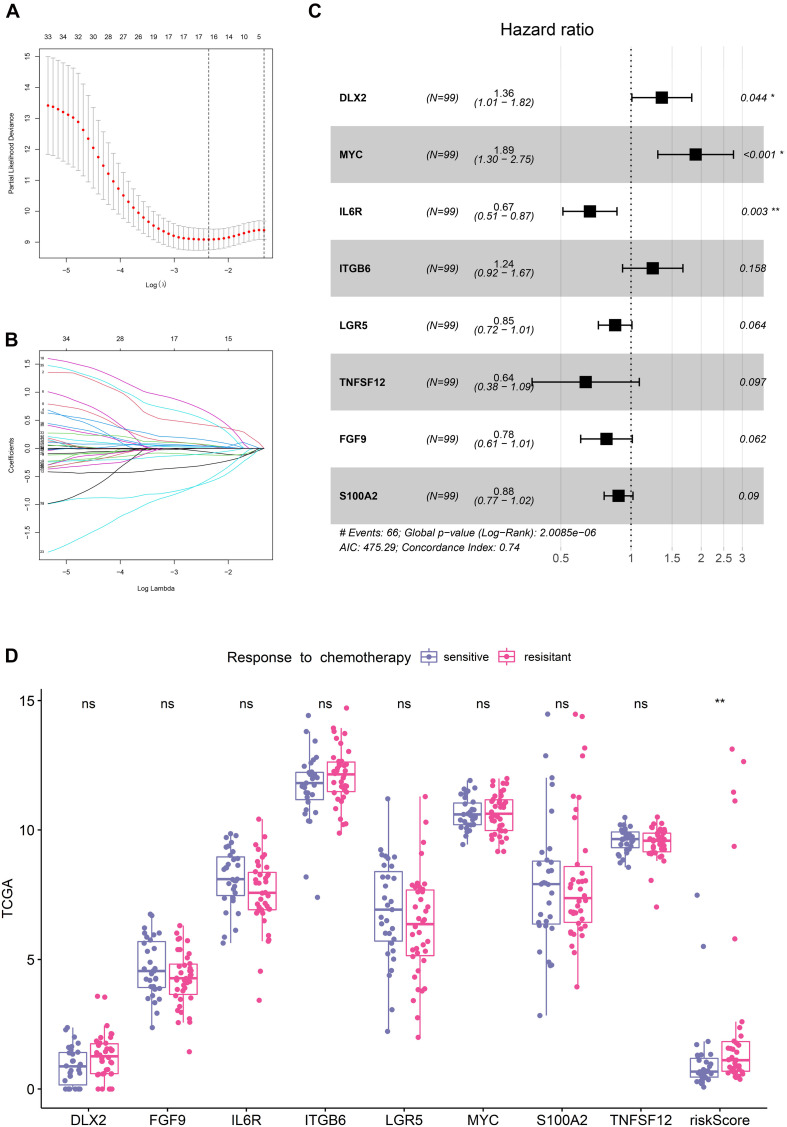
Establishment of the EMT-related gene signature in TCGA training cohort. **(A)** Cross-validation for tuning parameter (lambda) screening in the LASSO regression model. **(B)** LASSO coefficient profiles of 35 prognostic EMT-related genes. **(C)** Forest plot of the eight EMT-related genes. **(D)** Distribution of the eight genes and risk scores in patients stratified by the chemotherapy sensitivity.

With the selection criteria of *p* < 0.01, a total of 35 credibly prognostic EMT-related genes were identified through the univariate cox regression analysis in the TCGA training cohort. The LASSO regression algorithm was subsequently applied, and 16 candidate genes with most powerful predictive features were screened (Figures 1A,B). Then, multivariate cox regression analysis was performed on the 16 genes to avoid overfitting, and it finally determined an optimal 8-gene signature for DFS prediction (Figure 1C). Based on the expression levels and corresponding coefficients of these eight genes, we constructed a risk-score formula: Risk score = (0.30407 × expression value of DLX2) − (0.24245 × expression value of FGF9) − (0.40586 × expression value of IL6R) + (0.214597 × expression value of ITGB6) − (0.15683 × expression value of LGR5) + (0.638384 × expression value of MYC) − (0.12315 × expression value of S100A2) − (0.44785 × expression value of TNFSF12). K-M analysis illustrated that these eight individual genes adequately captured the DFS differences between low- and high-expression groups in the TCGA cohort (Supplementary Figure 1). In addition, the risk scores of chemotherapy-resistant patients were significantly higher than those of chemotherapy-sensitive patients, indicating the hazardous role of the signature (Figure 1D).

### Predictive Performance of the EMT-Related Gene Signature in TCGA Training Cohort

**FIGURE 2:**
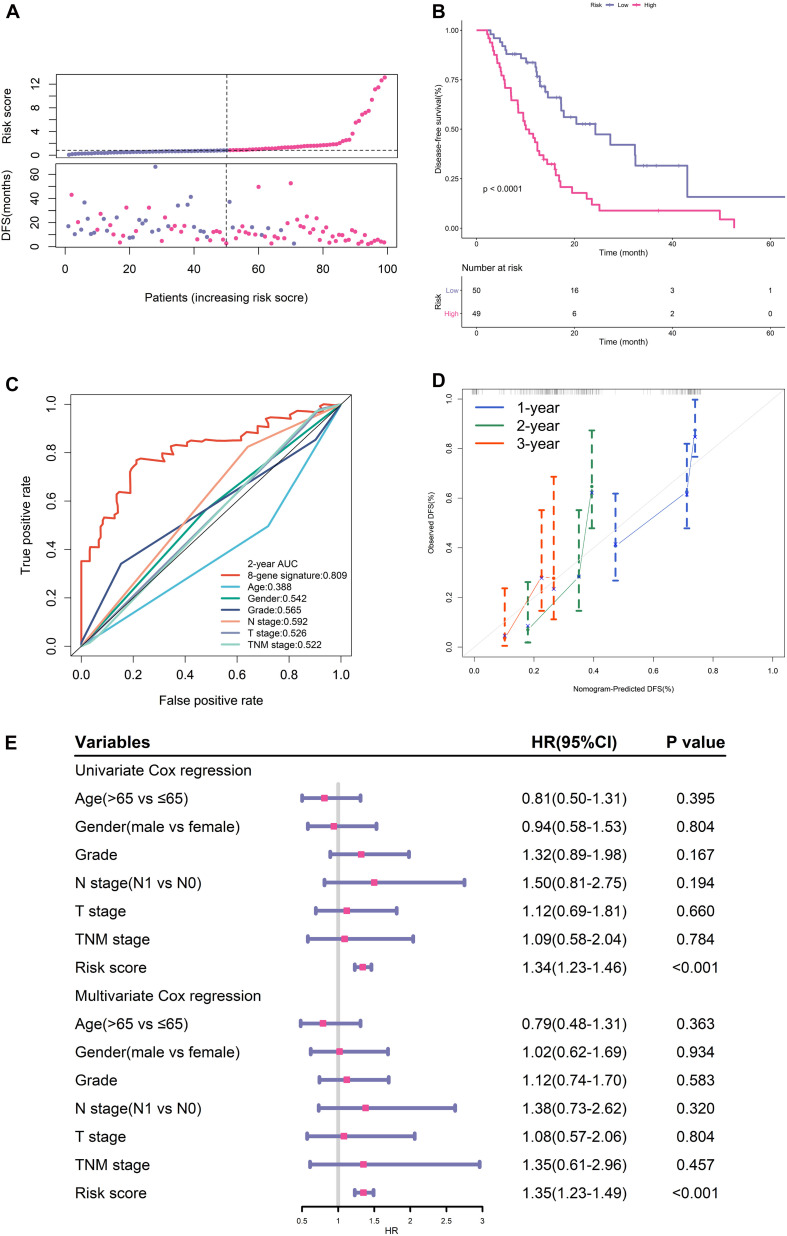
Prognostic performance of gene signature in TCGA training cohort. **(A)** From top to bottom are the risk score distribution and survival status distribution. **(B)** K-M survival curve for risk score. **(C)** ROC curve of the risk signature and clinical predictors. **(D)** Calibration curves for risk score. **(E)** Univariate and multivariate cox regression analyses of clinical parameters and gene signature for DFS.

The distribution of the risk scores and survival status were shown in Figure 2A. The results suggested that patients in high-risk group had remarkably decreased DFS time.

K-M analysis illustrated that patients in the low-risk group had longer DFS (Figure 2B). ROC analysis demonstrated that this signature had high accuracy as the area under the curve (AUC) value was 0.809. What’s more, the AUC value of this signature was high than that of clinical predictors including histological grade and TNM stage (Figure 2C).

The calibration curves proved the good agreement between predicted DFS and observed DFS (Figure 2D). In addition, both univariate and multivariate cox regression analyses certified that the proposed EMT signature was an independent risk factor for DFS (Figure 2E).

### Predictive Performance of the EMT-Related Gene Signature in Two Validation Cohorts

**FIGURE 3:**
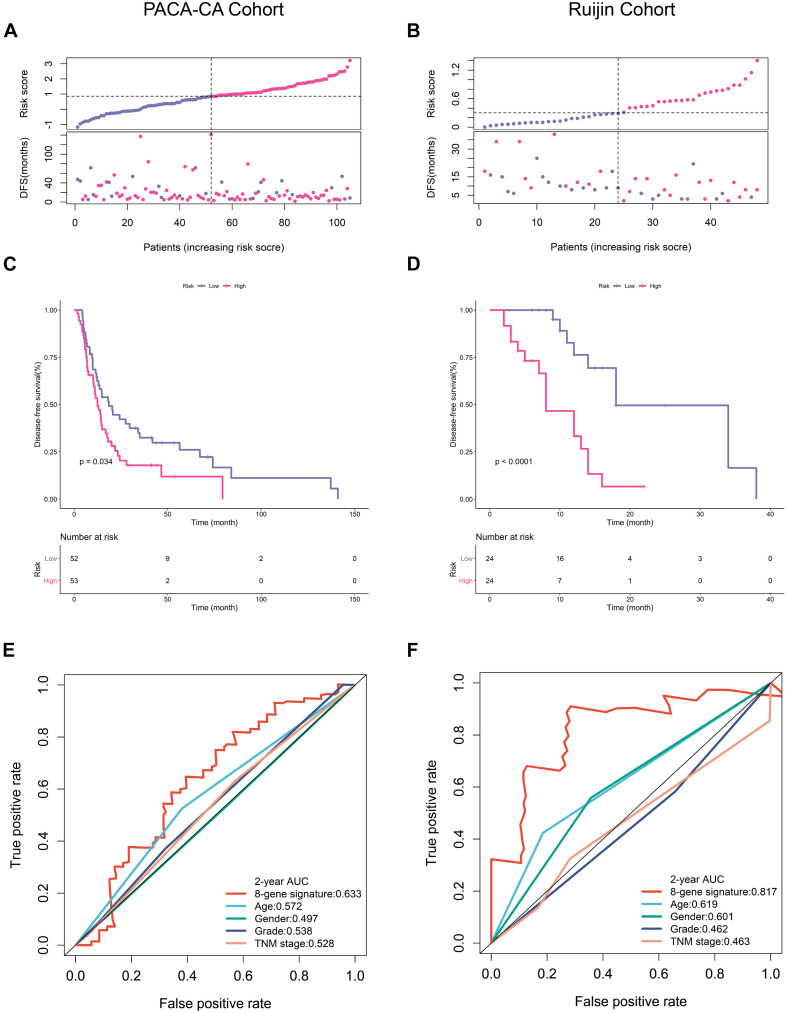
Prognostic validation in PACA-CA and Ruijin cohorts. **(A,B)** Distribution of risk score and survival status in PACA-CA and Ruijin cohorts, respectively. **(C,D)** K-M survival curves estimating DFS difference in two cohorts. **(E,F)** ROC curves of risk signature and clinical indicators in two cohorts.

We next verified the predictive accuracy of this signature in another public PDAC cohort (PACA-CA) and our own cohort (Ruijin). Figures 3A,B showed the distribution of the risk scores and survival status in these two cohorts. We observed that patients with a high-risk score had markedly increased recurrence rates. K-M survival curves estimated significantly decreased DFS of high-risk patients in both PACA-CA and Ruijin cohorts (Figures 3C,D). ROC curves demonstrated that this signature outperformed clinical indicators in predicting DFS (Figures 3E,F).

### Subgroup Analyses of the EMT-Related Gene Signature

**FIGURE 4:**
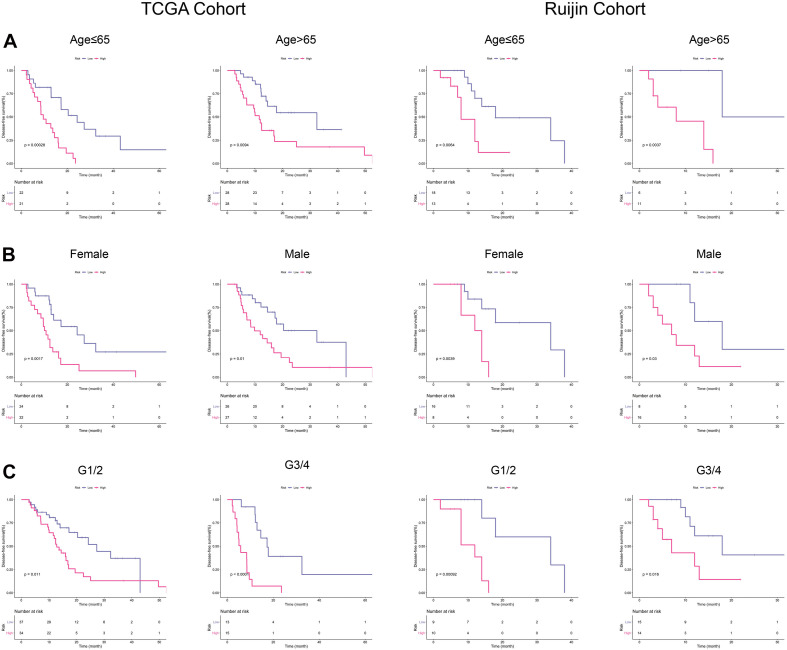
Subgroup analyses in TCGA and Ruijin cohorts. **(A)** K-M curves for the risk signature in patients stratified by age. **(B)** Survival difference in high- and low-risk patients stratified by gender. **(C)** K–M curves evaluating the DFS between low- and high-risk patients stratified by histological grade.

With the purpose to investigate the stability of this signature, we conducted subgroup analyses. As a small percentage of patients in PACA-CA cohort did not have clinical information regarding histological grade, we thus selected TCGA and Ruijin cohorts for further analyses. K-M curves showed that our signature had high-efficiency to distinguish patients with different DFS in every subgroup divided by age (Figure 4A), gender (Figure 4B), and histological grade (Figure 4C).

### Relationship Between Risk Score and Response to Chemotherapeutic Regimes

**FIGURE 5:**
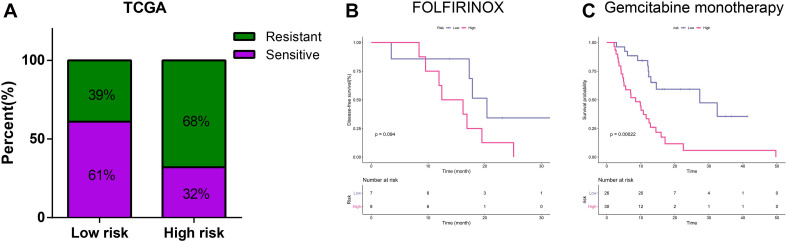
Relationship between risk score and chemotherapeutic regimes. **(A)** Relationship of risk score and chemotherapy sensitivity. **(B)** K-M curves for the risk signature in patients receiving FOLFIRINOX. **(C)** K-M curves for the risk signature in patients receiving gemcitabine monotherapy.

Figure 5A illustrated that PDAC patients with a low-risk score had higher response rates to adjuvant chemotherapy than patients with a high-risk score in TCGA training cohort (61 vs. 32%, *p* < 0.001). Currently, adjuvant chemotherapy in PDAC is based on few regimes. Gemcitabine remains the most effective monotherapy and is often applied to patient who are ineligible for more aggressive treatments (Oba et al., 2020; Turpin et al., 2020). As for patients in good status, the polychemotherapy regimen including fluorouracil, leucovorin, irinotecan, and oxaliplatin (FOLFIRINOX) is preferentially recommended in the adjuvant settings (Marabelle et al., 2020). Among samples receiving FOLFIRINOX chemotherapy, we found that patients in low-risk group had a longer DFS, although the difference was not statistically significant probably due to the limited sample size (Figure 5B). For samples receiving gemcitabine monotherapy, patients with a low-risk score had a significantly longer DFS (Figure 5C).

### Annotated Functions and Enriched Pathways Associated With the EMT-Related Gene Signature

**FIGURE 6:**
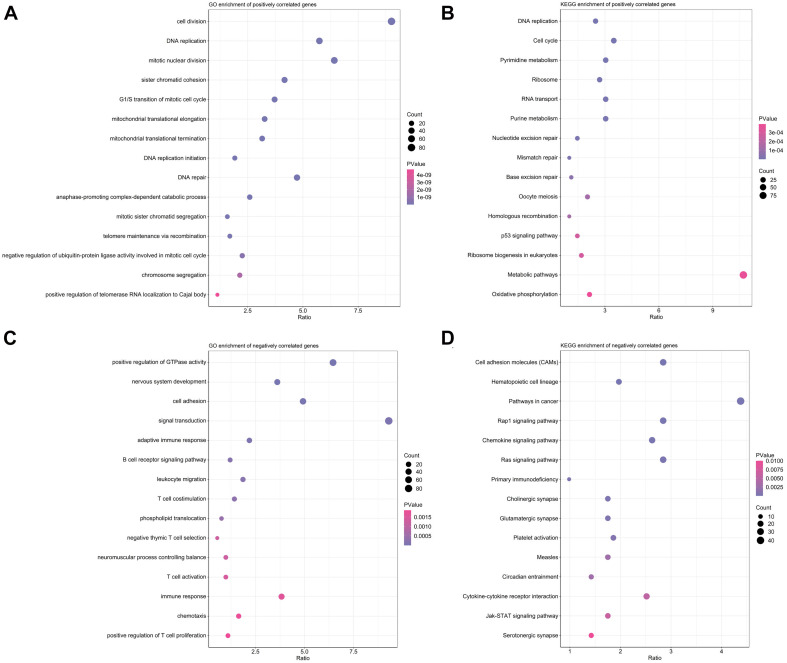
Function annotation and pathway enrichment analyses in TCGA training cohort. **(A,B)** Top 15 enriched biological processes in GO analysis **(A)** and pathways in KEGG analysis **(B)** for genes positively correlated with risk scores. **(C,D)** Top 15 enriched biological processes in GO analysis **(C)** and pathways in KEGG analysis **(D)** for genes positively correlated with risk scores.

As illustrated in Figures 6A,B, positively correlated genes with risk scores were mainly involved in pathways associated with response to treatment, such as DNA repair, DNA replication, cell cycle and mismatch repair. Genes negatively correlated with risk scores were closely associated with several immunological pathways like adaptive immune response, T cell costimulation, chemotaxis and chemokine signaling pathways (Figures 6C,D).

### Relationship Between Risk Scores and Expression Levels of Immune Check Points

**FIGURE 7:**
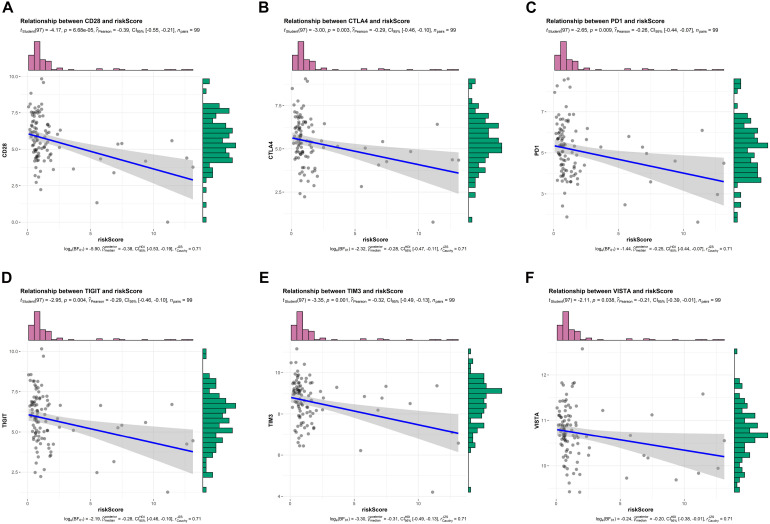
Relationship between risk scores and expression levels of immune checkpoints in TCGA training cohort. **(A–F)** Relationship between risk scores and CD28, CTLA4, PD1, TIGIT, TIM3, and VISTA, respectively.

Above findings suggested that risk scores were inversely correlated with T cell co-stimulation and immune response, so we wonder whether this signature could also predict response to ICIs. Recently, ICIs-based immunotherapy has drastically increased patient survival in certain cancers, but it is ineffective in the vast majority of patients with PDAC (Leinwand and Miller, 2020), biomarkers predicting response to ICIs thus are important for personalized oncology. As shown in Figures 7A–F, we observed that risk scores were negatively correlated with several common immune checkpoints, including CD28, CTLA4, PD1, TIGIT, TIM3, and VISTA. These findings indicated that PDAC patients who were not predicted to be sensitive to chemotherapy by our signature might be unsuitable for ICIs-based immunotherapy.

## Discussion

PDAC is a very devastating disease with extremely poor outcomes. As we all know, chemotherapy failure is one of the major problems to cure this disease and improve patient survival. PDAC features a notable intra- (Yachida and Iacobuzio-Donahue, 2013) and inter-tumoral (Cancer Genome Atlas Research Network, 2017) heterogeneity that drives chemoresistance. As cancer treatment has entered into the area of precision medicine, personalized therapy is a very attractive and laudable strategy. Determining the most effective drug to treat each patient with well balance between potential side events and expected survival benefits is definitely helpful to achieve the most favorable outcome. However, compared with other cancers, personalized treatments that translate the increased understanding of tumor molecular profiles into the clinical management are in their relative infancy for PDAC (Santofimia-Castaño and Iovanna, 2021).

Molecular characterization and subtyping of PDAC is providing a unique insight into predictive biomarkers for individualized treatments. The transcriptomic data has been a practical tool for PDAC subtyping and multiple stratification systems have been proposed to date by analyzing the transcriptional networks (Collisson et al., 2019). In addition, combining transcriptomic data with genomic sequencing, mutational landscape, immune infiltrate or genetic alteration can identify additional subtypes with clinical relevance (Bailey et al., 2016; Connor et al., 2017; Brunton et al., 2020; Rashid et al., 2020). More importantly than predicting patient prognosis and disease aggressiveness, recent studies find that transcriptomic data is also good at predicting chemotherapy sensitivity for PDAC (Deng et al., 2020; Nicolle et al., 2021; Nishiwada et al., 2021).

In this study, we initially analyzed the prognostic potential of EMT-related genes in predicting DFS through univariate cox regression analysis. Subsequently LASSO regression analysis and multivariate cox regression analysis identified an 8-gene signature for predicting response to adjuvant chemotherapy. K-M survival curves, ROC curves and calibration curves collectively proved the moderate accuracy of the signature in predicting DFS. Functional analysis indicated that this signature was closely related to several cancer-related pathways. Subgroup analysis demonstrated the cross-clinicopathology stability. Intriguingly, except from chemotherapy, the signature also had great potential to predict response to ICIs. In other words, patients with a high-risk score predicted by our signature were very likely to be insensitive to neither chemotherapy nor ICIs-based immunotherapy. In this way, high-risk patients might be exempted from unnecessary drug toxicity and heavy finical burden.

The EMT program plays an indispensable role in therapeutic resistance in cancers. Mechanically, it inhibits multiple apoptotic signaling pathways, enhances drug efflux, and gives rise to cancer stem cells. These all contribute to cancer cells’ increased resistance to anti-cancer drugs. In addition, EMT also upregulates several pathways that allow cancer cells to stave off the lethal effects of cytotoxic T cells, thus enhances resistance to immunotherapy (Huang et al., 2007). Transcriptional prognostic signatures based on EMT-related genes have been extensively reported recently (Cao et al., 2020; Zhang et al., 2020; Zhong et al., 2020; Cheng et al., 2021), but are rare in PDAC. Our study firstly constructed a robust response prediction model based on the eight EMT-related genes. We validated this signature in two public cohorts including American and Canadian populations, and our local cohort of Asian population, which enhanced the reliability and clinical applicability of this signature.

Despite explicit validation and considerable clinical relevance, this work is still based on retrospective data and has many limitations. Firstly, the cohorts used in this study are relatively small probably due to the low curative resection rates for PDAC patients.

Predictive efficiency needs to be verified in more prospective studies and larger cohorts. Second, owing to the limited sample size, some subgroup analyses cannot be implemented. For instance, 89 of 99 samples in TCGA cohorts are at stage II, subgroup analysis on tumor stage is thus meaningless. Third, detailed chemotherapy regimens are largely unknown in PACA-CA cohorts and incomplete in TCGA cohorts. Fourth, more *in vivo* and *in vitro* experiments are needed to elucidate biological function of eight genes in PDAC progression.

In conclusion, we proposed an EMT-related gene signature with satisfactory performance in predicting response to adjuvant chemotherapy. Functionally, it was associated with cell cycle, DNA repair and DNA replication. The signature outperformed clinical indicators in predictive chemotherapy sensitivity. After all, this signature was based on the retrospective cohorts and needed to be further validated in more prospective cohorts.

## Data Availability Statement

The original contributions presented in the study are included in the article/Supplementary Material, further inquiries can be directed to the corresponding author/s.

## Ethics Statement

The studies involving human participants were reviewed and approved by the Ethics Committee of Ruijin Hospital affiliated with Shanghai Jiao Tong University. The patients/participants provided their written informed consent to participate in this study.

## Author Contributions

ZF and CP designed the study and wrote the manuscript. ZF, KL, and JL participated in data analysis, discussion, and language editing. YW reviewed the manuscript. All authors contributed to the article and approved the submitted version.

## Conflict of Interest

The authors declare that the research was conducted in the absence of any commercial or financial relationships that could be construed as a potential conflict of interest.

## Footnotes

1 https://tcga.xenahubs.net

2 https://icgc.org/

## Abbreviations

AUC: area under the curve
DFS: disease-free survival
EMT: epithelial to mesenchymal transition
GOm: Gene Ontology
ICGC: International Cancer Genome Consortium
ICIs: Immune-checkpoint inhibitors
KEGG: Kyoto Encyclopedia of Genes and Genomes (KEGG)
K-M: Kaplan-Meier
PDAC: pancreatic ductal adenocarcinoma
QRT-PCR: quantitative real time polymerase chain reaction
ROC: receiver operating characteristic
TCGA: The Cancer Genome Atlas
TNM: tumor node metastasis.
