# Supplementary material for: An EMT-Related Gene Signature for Predicting Response to Adjuvant Chemotherapy in Pancreatic Ductal Adenocarcinoma
Source: Front Cell Dev Biol. 2021 Apr 30;9:665161. doi: 10.3389/fcell.2021.665161 (PMC8119901; doi:10.3389/fcell.2021.665161)
Supplement: Supplementary file 1 [file Table_1.DOCX]

| **Name** | **Primer (5'-3')** |
| --- | --- |
| **DLX2-F** | **ATGCACTCGACCCAGATCG** |
| **DLX2-R** | **GGCTTGGTACTGGTAGGAACC** |
| **FGF9-F** | **ATGGCTCCCTTAGGTGAAGTT** |
| **FGF9-R** | **CCCAGGTGGTCACTTAACAAAAC** |
| **IL6R-F** | **CATGTGCGTCGCCAGTAGT** |
| **IL6R-R** | **AGCTCAAACCGTAGTCTGTAGA** |
| **ITGB6-F** | **TCCATCTGGAGTTGGCGAAAG** |
| **ITGB6-R** | **TCTGTCTGCCTACACTGAGAG** |
| **LGR5-F** | **CTCCCAGGTCTGGTGTGTTG** |
| **LGR5-R** | **GAGGTCTAGGTAGGAGGTGAAG** |
| **MYC-F** | **GGCTCCTGGCAAAAGGTCA** |
| **MYC-R** | **CTGCGTAGTTGTGCTGATGT** |
| **S100A2-F** | **GCCAAGAGGGCGACAAGTT** |
| **S100A2-R** | **AGGAAAACAGCATACTCCTGGA** |
| **TNFSF12-F** | **GAGGGGAAGGCTGTCTACCT** |
| **TNFSF12-R** | **GAACCTGGAAGAGTCCGAAGTA** |
| **GAPDH-F** | **GCACCGTCAAGGCTGAGAAC** |
| **GAPDH-R** | **TGGTGAAGACGCCAGTGGA** |

**Supplementary Table S1. Quantitative real-time PCR primer sequences.**
