# Supplementary figures and images for: An EMT-Related Gene Signature for Predicting Response to Adjuvant Chemotherapy in Pancreatic Ductal Adenocarcinoma
Source: Front Cell Dev Biol. 2021 Apr 30;9:665161. doi: 10.3389/fcell.2021.665161 (PMC8119901; doi:10.3389/fcell.2021.665161)

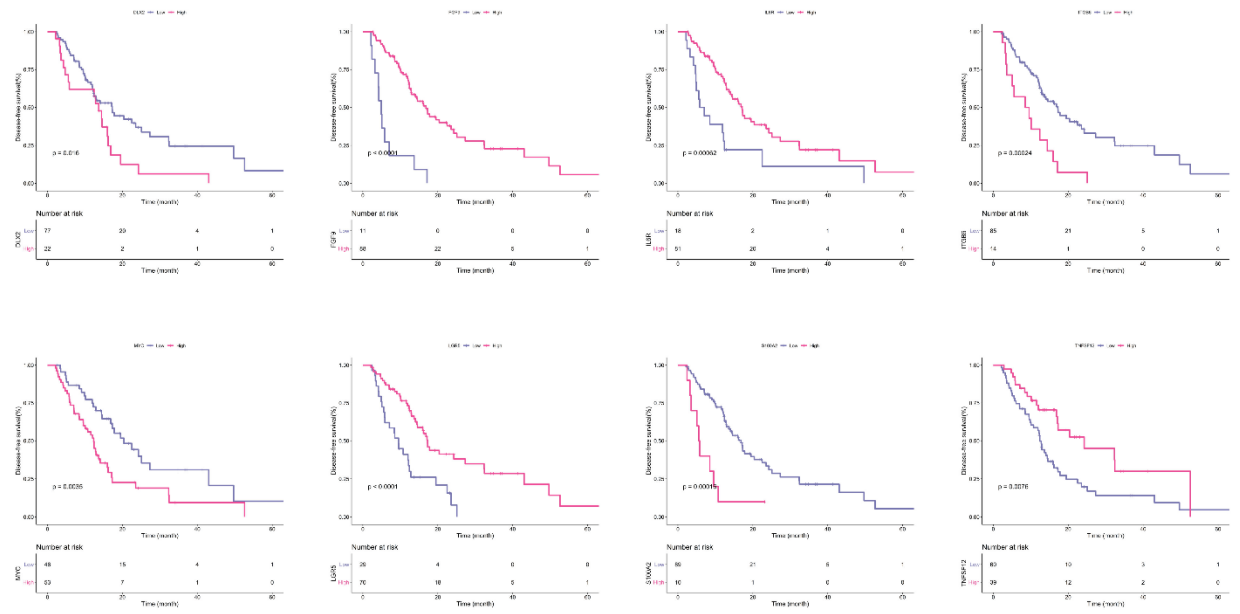

**Supplementary Figure. S1 K-M curves of the eight genes.**

Supplement: Supplementary file 2 [file Data_Sheet_1.PDF]
